# Effects of Microminerals on Performance and Metabolic Adaptation in Heat-Stressed Dairy Cows: A Review

**DOI:** 10.1007/s12011-025-04911-8

**Published:** 2025-11-17

**Authors:** M. R. Rezaei-Ahvanooei, M. Lamanna, R. Colleluori, A. Formigoni, M. A. Norouzian, A. Assadi-Alamouti, D. Cavallini

**Affiliations:** 1 Department of Animal and Poultry Science, College of Agricultural Technology, University of Tehran, Tehran, 3391653755 Iran; 2 Department of Veterinary Medical Sciences, University of Bologna, Ozzano dell’Emilia, 40064 Italy

**Keywords:** Dairy cows, Heat stress, Metabolic adaptation, Microminerals, Production performance

## Abstract

This narrative review summarizes the role of microminerals in mitigating heat stress (HS) in dairy cows, with a focus on antioxidant defense, immune function, and metabolic regulation. A systematic literature search (2000–2025) in Scopus, Google Scholar, and PubMed included only studies conducted on dairy cows under HS. Evidence indicates that chromium, selenium, zinc, copper, cobalt, and combined micromineral supplements affect production performance, physiological responses, and overall health in heat-stressed dairy cattle. Chromium supplementation has been widely studied for its role in enhancing energy metabolism, contributing to increased dry matter intake (DMI), milk yield, and better heat tolerance. Selenium, as part of selenoproteins, supports antioxidant capacity, thermotolerance, and immune function, though its effect on milk yield remains inconsistent. Zinc helps antioxidant defense, reduces inflammation, and maintains calcium homeostasis, but its impact on production outcomes is unclear. Copper supports immune and antioxidant functions, with results influenced by dose, source, and animal physiological status. Cobalt, essential for vitamin B12 synthesis and rumen microbial activity, is linked to higher milk and milk fat production; however, excessive intake may impair milk protein synthesis. Multi-micromineral strategies show synergistic benefits, including improved milk composition, udder health, immune function, and reproductive performance under HS. Nevertheless, responses vary with mineral form, dosage, physiological stage, and environmental conditions. In conclusion, micromineral supplementation represents a promising nutritional strategy to alleviate HS in dairy cattle. Yet, variability in outcomes highlights the need for further research to define optimal dosing, clarify mineral interactions, and establish practical guidelines for sustainable productivity and animal welfare in heat-stressed systems.

## Introduction

Heat stress (HS) poses a significant challenge for dairy cattle, particularly in regions experiencing elevated ambient temperatures during the summer [1]. HS is broadly defined as the cumulative effect of external factors that elevate an animal’s core body temperature, thereby initiating a cascade of physiological adaptations [2]. Collier et al. [3] reported that dairy cows experience HS when the Temperature-Humidity Index (THI) surpasses 68, resulting in a significant decline in both physiological function and productive performance. Elevated ambient temperature and humidity adversely affect livestock physiology, leading to reduced feed intake and productivity [4], while exacerbating inflammation and oxidative stress, particularly when animals cannot efficiently dissipate excess heat [5]. Moreover, HS in dairy cows is a significant contributor to reproductive disorders, further compromising overall productivity and herd sustainability [6].

Dietary interventions, including micromineral supplementation, enhance metabolic function [7–9], mitigate oxidative stress [9–12], reduce pro-inflammatory cytokines [11, 13, 14], improve reproductive performance [9, 15, 16], strengthen gut barrier integrity [17], and ultimately enhance overall productivity [7, 12, 18–21] in heat-stressed dairy cows. Meanwhile, organic chromium (Cr) has been extensively studied for its diverse physiological effects, demonstrating a significant role in metabolic regulation, immune system enhancement, and overall performance optimization in heat-stressed dairy cows. Empirical evidence suggests that Cr supplementation effectively lowers the body temperature of dairy cows, thereby mitigating the adverse impacts of HS [11, 12, 20, 22]. Accordingly, the regulation of body homeostasis enables the dairy cow to enhance feed intake, aligning with the findings of our literature review [7, 11, 12, 18, 19, 23]. By enhancing the binding of insulin to extracellular receptors, Cr facilitates the mobilization of the insulin-dependent glucose transporter type 4 (GLUT4), thereby increasing cellular glucose uptake [24, 25]. The enhancement of glucose metabolism resulting from Cr supplementation may also have a beneficial impact on reproductive performance [15, 26]. Accordingly, Cr supplementation can enhance dairy cow productivity under HS by stabilizing body homeostasis, stimulating feed intake, and optimizing energy utilization efficiency. Selenium (Se), known for its essential role in enhancing antioxidant defense under oxidative stress [8–10, 27], has been widely investigated in both organic (e.g., Se-yeast or hydroxy-selenomethionine) and inorganic (sodium selenite) forms for its potential to mitigate HS in dairy cows. Selenium is essential for the synthesis of glutathione peroxidase [28] and, in addition to enhancing antioxidant status, it modulates metabolism [8, 9], strengthens the immune system [8], and may improve reproductive performance [9] in dairy cows under HS. However, Se does not have a significant direct effect on feed intake or milk production [10, 29–31]. Research on the effects of other microminerals, such as zinc (Zn), copper (Cu), and cobalt (Co) in dairy cows under HS remains limited, and the existing data are insufficient to draw definitive conclusions.

The purpose of this review is to provide a comprehensive evaluation of the role of microminerals, such as Cr, Se, Zn, Cu, and Co, in mitigating the adverse effects of HS on dairy cows. By synthesizing the available data, this article aims to clarify the potential benefits and limitations of micromineral supplementation in improving antioxidant status, immune function, metabolism, and overall productivity in heat-stressed dairy cows. Additionally, this review seeks to identify gaps in the current research and highlight areas that require further investigation to optimize HS management in dairy production systems.

## Literature Search Strategies

**Fig. 1:**
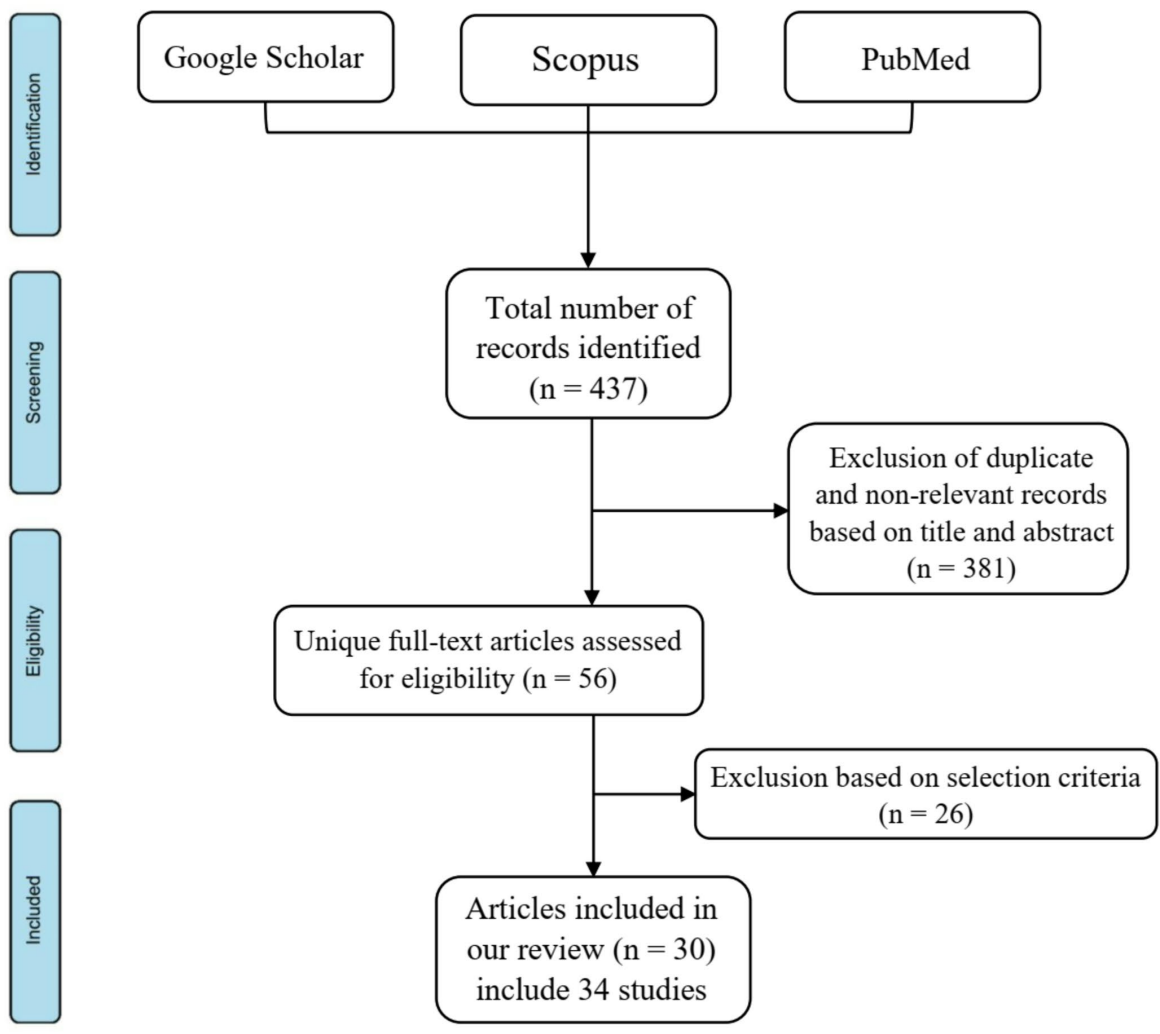
Flowchart of the literature search, identification, and screening process for selecting suitable studies (search conducted during January and February 2025)

**Fig. 2:**
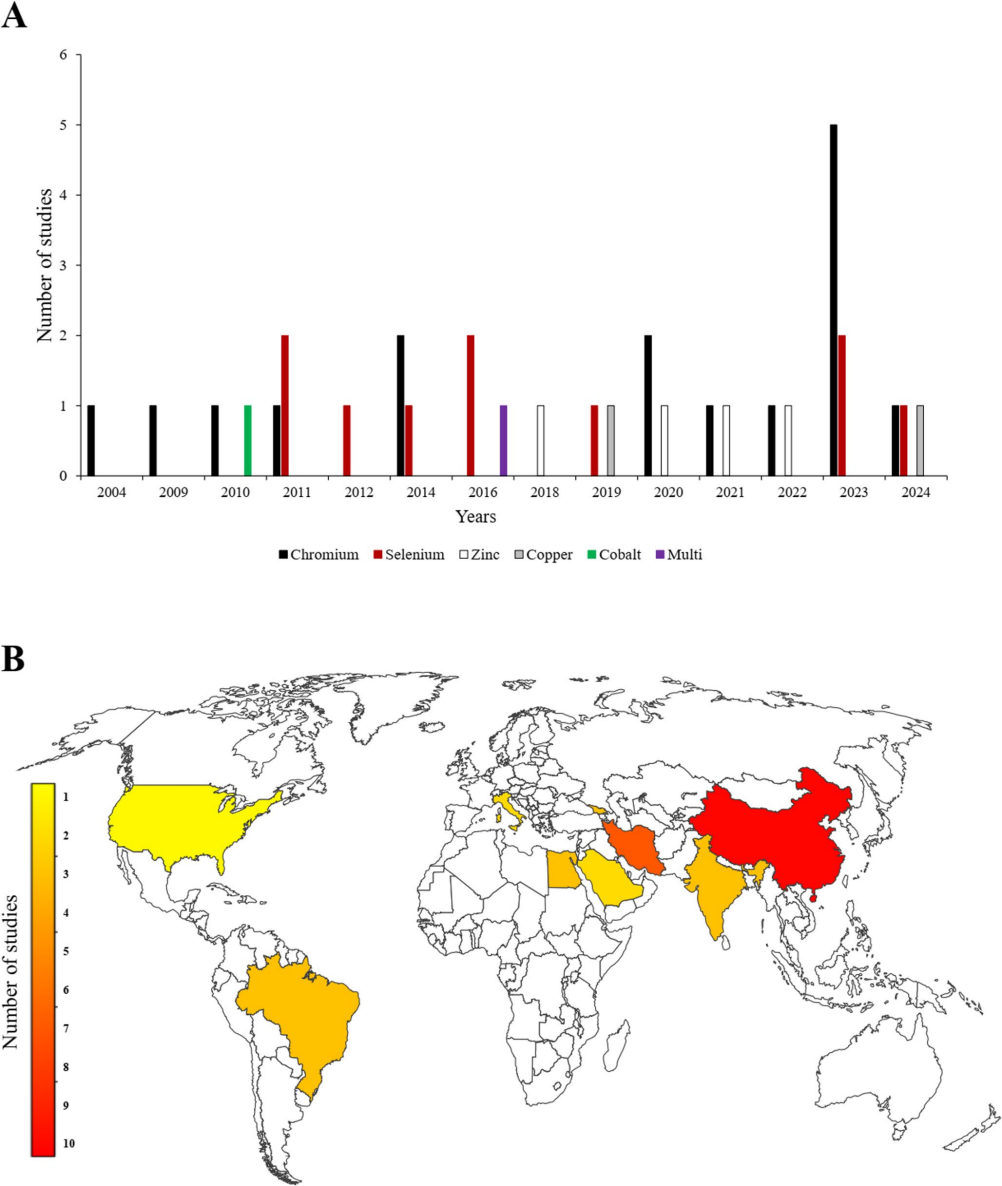
Publication timeline of studies across different years for each micromineral (Panel **A**), and the geographical distribution of the studies included in this literature review (Panel **B**)

This study is a narrative review that examines the effects of microminerals on dairy cows under HS. A systematic literature search was conducted in three major academic databases-Scopus, Google Scholar, and PubMed-during January and February 2025. The search strategy employed a comprehensive set of keywords and their combinations, including “micromineral”, OR “microminerals”, OR “microelement”, OR “microelements”, OR “trace element”, OR “trace elements”, OR “Chromium”, OR “Cr”, OR “Selenium”, OR “Se”, OR “Zinc”, OR “Zn”, OR “Copper”, OR “Cu”, OR “Cobalt”, OR “Co”, OR “Manganese”, OR “Mn”, OR “Iodine”, OR “I”, OR “Molybdenum”, OR “Mo”, OR “Iron”, OR “Fe”, AND “dairy cow”, OR “dairy cows”, OR “dairy cattle”, OR “livestock”, OR “ruminants”, OR “animals”, AND “heat stress”, OR “thermal stress”, OR “hot season”, OR “summer”. One of the inclusion criteria was that the selected studies had to be peer-reviewed publications from 2000 to 2025, with no language restrictions. Initially, 437 articles were identified, of which 381 were deemed duplicate and irrelevant following a preliminary title-based screening and subsequently excluded. The remaining 56 articles were imported into EndNote for systematic organization and underwent a secondary screening based on title, abstract, and full-text assessment to ensure alignment with the study’s objectives. Ultimately, 30 articles (including 34 studies) meeting all inclusion criteria were selected for review (Fig. 1). It is important to state that one of the selection criteria for articles included in this review was the requirement that each study conducted comparisons exclusively under HS conditions. Consequently, all studies and data incorporated in this review focus primarily on the effects of the specified microminerals on dairy cows subjected to HS. Comparisons within each study were made solely within the same environmental context that is, HS without including comparisons between heat-stressed cows and those maintained under thermoneutral conditions. These studies focused on the effects of chromium, selenium, zinc, copper, cobalt, and multi-micromineral supplementation in heat-stressed dairy cows, whereas the roles of other microminerals in this context remain largely unexplored. Figure 2 presents the publication timeline of articles across different years for each micromineral along with the geographical distribution of the studies included in this literature review. Further details on the selected articles are presented in Table 1. To maintain the study’s relevance and comprehensiveness, continuous monitoring of database updates was performed throughout the writing process to incorporate newly published, pertinent research.

**Table 1:** Summary of peer-reviewed studies evaluating the effects of micromineral supplementation on dairy cows under heat stress (HS)

Articles	Country	Mineral	Source	Dosage (mg/d/cow)^1^	T. Number of cows	Breed	THI^2^	DIM at the start^3^	Exp. Duration^4^	Parity^5^	Changes in outcomes^6^
Al-Saiady et al. 2004 [18]	Saudi Arabia	Chromium	Organic (chromium yeast)	4 g/d/cow of Cr-yeast (approximately 12 mg/d/cow)	160	Holstein	78.6	125	70	M	DMI ↑, Milk Y ↑ Globulin ↓, Cholesterol ↑, TP ↓, Alb/Globulin ↑
An-Qiang et al. 2009 [19]	China	Chromium	Organic (chromium picolinate)	3.6, 7.2, 10.8	24	Holstein	79.61	20	63	M	**For 3.6 mg**: DMI ↑ **For 7.2 mg**: DMI ↑, Milk Y ↑, FCM ↑ Glu ↑, Glu/Insulin ↑, CK ↓ **For 10.8 mg**: DMI ↑, Milk Y ↑, FCM ↑ Glu ↓, Glu/Insulin ↑, CK ↓
Soltan 2010 [7]	Saudi Arabia	Chromium	Organic	6	120	Holstein	95.5	− 21	105	NR^7^	DMI ↑, Milk Y ↑ **Measured in DIM 28**: NEFA ↓, Cortisol ↓, Insulin ↑
Mirzaei et al. 2011 [23]	Iran	Chromium	Organic (Cr-methionine)	31, 62	15	Holstein	77.7	38	49	M & P	**For 31 mg**: DMI ↑, ECM ↑, Milk FY ↑, Milk P ↑, Milk PY ↑, Milk TS ↑, Milk TS Y ↑, BW change ↓ Insulin ↓, Insulin/glucagon ↓, Alb ↑, Alb/globulin ↑,NEFA ↓ **For 62 mg**: DMI ↑, Milk P ↑ Insulin/glucagon ↓, Alb/globulin ↑
Zhang et al. 2014a [45]	China	Chromium	Organic (chromium picolinate)	3.5	24	Holstein	73.9	115	15	P	No significant effect on physiological parameters, serum metabolites, antioxidant and inflammatory indicators (Except Cholesterol ↑)
Zhang et al. 2014b [45]	China	Chromium	Organic (chromium picolinate)	3.5	24	Holstein	80.3	115	24	P	Cholesterol ↓, IL-10 ↑, Hsp72 ↑
Ribeiro et al. 2020 [33]	Brazil	Chromium	Organic (chromium picolinate)	9.1	36	Girolando	85	71	11	NR	No significant effect on performance, reproductive parameters, and serum metabolites
Shan et al. 2020 [11]	China	Chromium	Organic (chromium yeast)	3.1, 6.2, 9.3	24	Holstein	> 72	105	56	M	DMI ↑, RR ↓, RT ↓ GSH-Px ↑, SOD ↑, TAC ↑, MDA ↓, IgG ↑, IL-1β ↓, IL-2 ↓, IL-4 ↓, IL-10 ↓, Chromium concentration ↑
do Rego Leal et al. 2021 [42]	Brazil	Chromium	Organic (chromium yeast)	20.5	46	Holstein	> 72	207	35	NR	No significant effect on milk yield and serum metabolites (Except Cholesterol ↓)
Sultana et al. 2022 [22]	India	Chromium	Organic (chromium propionate)	6.6	24	Holstein Friesian	77.5	56	90	P	RT ↓, RR ↓ TP ↑, Alb ↑, Alb/globulin ↑
Zhao et al. 2023 [13]	China	Chromium	Organic (chromium propionate)	12.5	15	Holstein	73.2	32	31	M	Milk Y ↑ TNF-α ↓, IL-1β ↓, IL-6 ↓, IL-10 ↓ **Ruminal VFAs**: Acetate ↑, Propionate ↑, Butyrate ↑
Soffa et al. 2023 [15]	USA	Chromium	Organic (chromium propionate)	12	22	Holstein	≥ 68	20	24	M	polymorphonuclear leukocyte ↓, Diameter of 6–9 mm follicles ↑, progesterone/average corpus luteum volume ↑
Zhang et al. 2023 [34]	China	Chromium	Organic (chromium yeast)	8 g/d/cow of Cr-yeast (approximately 24 mg/d/cow)	24	Holstein	79.4	− 27	30	M	**Nutrient digestibility**: Ether extract ↑ Milk Butterfat ↑, Milk SNF ↓ Cortisol ↓, Norepinephrine ↓ TAC (prepartum) ↑, GSH-Px ↑
Wang et al. 2023 [12]	China	Chromium	Organic (chromium propionate)	4, 8, 12	64	Holstein	> 72	143	49	M	**For 4 mg**: DMI ↑, Milk Y ↑, MUN ↓, SCC ↓ RR ↓ GSH-Px ↑, SOD ↑, BUN ↓ **Ruminal VFAs**: Acetate ↑ **For 8 mg**: DMI ↑, Milk L ↑ RT ↓ SOD ↑, BUN ↓ **Ruminal VFAs**: Acetate ↑ **Nutrient digestibility**: NDF ↑, Fecal Cr ↑ **For 12 mg**: Milk L ↑ BUN ↓ **Nutrient digestibility**: Chromium retention ↑
Wo et al. 2023 [20]	China	Chromium	Organic (chromium yeast)	6.2	12	Holstein	79	125	56	M	Milk Y ↑, Milk P ↑, Milk L ↑, Milk PY ↑, Milk LY ↑, Milk TS ↑ RT ↓ Glu ↑, BUN ↓, Insulin ↓, T4 ↑, T3 ↓, nicotinamide ↑
Shan et al. 2025 [17]	China	Chromium	Organic (chromium yeast)	6.2	12	Holstein	79	125	56	M	MCP ↑ **Relative abundances of bacterial genera in ruminal fluids**: Olsenella ↑, Enterobacter ↓, Escherichia-Shigella ↓, Oribacterium ↓, Bacteroidetes_BD2-2 ↓, Lachnospiraceae_UCG-002 ↑, Shuttleworthia ↑, **Abundance of ruminal amino acid**: D-(+)-proline ↓, DL-glutamic acid ↓, DL-lysine ↓, Gly-l-pro ↓, L-(-)-serine ↓, L-(+)-alanine ↓, L-(+)-aspartic acid ↓
Calamari et al. 2011a [8]	Italy	*Selenium*	Organic (Selenium yeast)	0.31 and 0.50 mg/kg DM	20	Friesian	> 72	110	140	M	**For 0.31 mg**: BHBA ↓, NEFA ↑, GPX-3 ↑, Thiobarbituric acid reactive substances (TBARS) ↓, Lymphocytes ↓ **For 0.5 mg**: BHBA ↓, NEFA ↑, Na ↓, GPX-3 ↑, Thiobarbituric acid reactive substances (TBARS) ↓, Lymphocytes ↓
Calamari et al. 2011b [8]	Italy	*Selenium*	inorganic (sodium selenite)	0.31 and 0.50 mg/kg DM	20	Friesian	> 72	125	140	M	**For 0.31 mg**: BHBA ↓, NEFA ↑, Na ↓, GPX-3 ↑, Lymphocytes ↓ **For 0.5 mg**: BHBA ↓, NEFA ↑, Na ↓, GPX-3 ↑, Lymphocytes ↓
Tahmasbi et al. 2012 [29]	Iran	*Selenium*	inorganic (sodium selenite), Injections every two weeks in the form of selenium-vitamin E	0.357 (or 5 mg every two weeks)	20	Holstein	73.2	86	98	M & P	No significant effect on performance, physiological parameters, serum metabolites, and rumen fluids (Except Milk L ↑)
Oltramari et al. 2014 [30], (Comparison only between two different Se sources)	Brazil	*Selenium*	Organic (Selenium yeast) and inorganic (sodium selenite)	5 (for both organic and inorganic)	24	Holstein Friesian & Brown Swiss	76	NR	124	NR	**Organic to inorganic Comparison**: Milk F ↑, SCC ↓, RR ↑, haircoat temperature ↓
Khalifa et al. 2016a [27]	Egypt	*Selenium*	inorganic (sodium selenite), Injections every two weeks in the form of selenium-vitamin E	1.2 and 2.4 (or 16.7 and 33.4 mg every two weeks)	15	Holstein	80	80	60	M	**For 1.2 mg**: No significant effect on performance, physiological parameters, and serum metabolites **For 2.4 mg**: Milk P ↑ GSH-Px ↑
Khalifa et al. 2016b [27]	Egypt	*Selenium*	Organic (Selenium yeast)	6.76	10	Holstein	80	80	60	M	SCC ↓ GSH-Px ↑
Sun et al. 2019 [10], (Comparison only between two different Se sources)	China	*Selenium*	Organic (hydroxy-selenomethionine) and inorganic (sodium selenite)	3.36 (for both organic and inorganic)	8	Holstein	83	141	9	M	**Set sodium selenite as control** Milk F ↓, Milk total Se ↑ Serum total Se ↑, Milk/serum of total Se ↑, TAC ↑, MDA ↓, Hydrogen peroxide ↓, Nitric oxide ↓, ALT ↓
Kumar et al. 2023a [31]	India	*Selenium*	Organic (Selenium yeast)	4 g/d Se + 4 g/d yeast	18	indigenous dairy cows	86.5	NR	45	M	Final BW ↑, Final BCS ↑ Cortisol ↑ Hemoglobin ↑, packed cell volume (PCV) ↑
Kumar et al. 2023b [31]	India	*Selenium*	Organic (Selenium yeast)	4 g/d Se + 4 g/d yeast	18	Crossbred dairy cows	86.5	NR	45	M	Final BCS ↑, Milk F ↑ Lipidperoxidase ↑
Shaarawy et al. 2024 [9]	Egypt	*Selenium*	inorganic (sodium selenite), Injections in the form of selenium-vitamin E	27.7 (just before AI), 27.7 (just at the AI time), and both	20	Friesian	> 77	55 to 70	35	M	**before AI**: Follicle Number (On the 10th day) ↑, Follicle Diameter (On the 10th day) ↑ Cortisol ↓, Prolactin ↑ MDA ↓,GSH ↑, CAT ↑, SOD ↑, TAC ↑ **at the AI time**: T3 ↑, Cortisol ↓, Prolactin ↑ MDA ↓, GSH ↑, CAT ↑, SOD ↑, TAC ↑ **both**: Follicle Number (On the 10th day) ↑, Follicle Diameter (On the 10th day) ↑ Progesterone ↑, T3 ↑, Cortisol ↓, Prolactin ↑ MDA ↓, GSH ↑, CAT ↑, SOD ↑, TAC ↑
Weng et al. 2018 [51], (Comparison of two different Zn sources)	Georgia	Zinc	Organic (Zn-Met) and inorganic (Zn-hydroxychloride)	Organic 0.773 and inorganic 1.57 g/d/cow	72	Holstein	77.8	100	84	M	No significant effect on performance and gene expression
Marins et al. 2020 [49], (Comparison of two different Zn sources)	Georgia	Zinc	Organic (Zn-Met) and inorganic (Zn-hydroxychloride)	Organic 0.773 and inorganic 1.57 g/d/cow	72	Holstein	77.8	100	84	M	**Organic to inorganic Comparison**: No significant effect on abundance of proteins associated with metabolism in mammary tissues and serum metabolites (Except serum Triglyceride concentration ↑)
Rivas et al. 2021 [50], (Comparison of two different Zn sources)	Georgia	Zinc	Organic (Zn-Met) and inorganic (Zn-hydroxychloride)	Organic 0.773 and inorganic 1.57 g/d/cow	72	Holstein	77.8	100	84	M	**Organic to inorganic Comparison**: Gene expression in mammary tissue: SOD2 ↓ Mammary cell apoptotic: Epithelium ↑, Total ↑
Danesh Mesgaran et al. 2022 [14]	Iran	Zinc	Organic (rumen-protected Zn-Met)	2.37 g/d/cow	62	Holstein	75.1	28	42	M	Serum concentration of zinc ↑, calcium ↑, haptoglobin ↓, IL-1β ↓, total antioxidant status ↑
Khodamoradi et al. 2019 [52]	Iran	*Copper*	Inorganic (copper sulfate), Injection on days − 40, − 20, 0 and 20 relative to calving	75	40	Holstein	> 65	− 40	130	M	No significant effect on performance, serum metabolites, and immune system indicators (Except red blood cell count at calving ↑)
Jafari et al. 2024 [53]	Iran	*Copper*	Inorganic (copper sulfate), Injection on days − 40, − 20, 0 and 20 relative to calving	75	40	Holstein	> 72	− 40	130	M	In DIM 30: Serum concentrations of Cu ↑, SOD ↑
Karkoodi 2010 [21]	Iran	Cobalt	Inorganic	30, 40, 50	12	Holstein	-	134	96	M	**For 30 mg**: Milk Y ↑, FCM ↑, Milk FY ↑, Milk PY ↑ **For 40 mg**: Milk Y ↑, FCM ↑, Milk FY ↑, Milk PY ↓ **For 50 mg**: Milk Y ↑, FCM ↑, Milk FY ↑, Milk PY ↓
Khorsandi et al. 2016 [16]	Iran	Multi	sustained-release multi-trace element/vitamin bolus (With the content of inorganic salts including copper oxide, sodium selenite, cobalt sulfate, potassium iodide, manganese sulfate, zinc oxide and zinc sulfate, vitamin A, vitamin D3, vitamin E)	the mean daily nutrient release rate was: 136.5 mg of Cu, 2.1 mg of Se, 2.0 mg of Co, 4.1 mg of I, 69.4 mg of Mn, and 111.5 mg of Zn	50	Holstein	71.43	− 21	240	M	**Composition of milk samples collected up to DIM 63**: Milk F ↑, Milk P ↑, Milk SNF ↑, SCC ↓ **Blood samples collected up to DIM 60**: TP (at calving) ↑ Days open (calving to conception) ↓,Cumulative pregnancy rate (5th AI) ↑

1. The unit of the values ​​mentioned is mg/day/cow unless another unit is written

2. Temperature Humidity Index

3. Days in milk (DIM) when starting supplementation

4. Total trial period from start of supplementation to end (days)

5. Parity includes Multiparous (M) and Primiparous (P)

6. Only differences with a significance level of *P* ≤ 0.05 are listed. Abbreviations: ↑ means increase, ↓ means decrease, (DMI): Dry matter intake, (Milk Y): Milk yield, (Milk F): Milk fat, (Milk FY): Milk fat yield, (Milk P): Milk protein, (Milk PY): Milk protein yield, (Milk L): Milk lactose, (Milk LY): Milk lactose yield, (Milk TS): milk total solids, (Milk TS Y): milk total solids yield, (Milk SNF): Milk solids-not-fat, (FCM): fat corrected milk, (ECM): Energy corrected milk, (SCC): Milk somatic cell count, (NDF): Neutral detergent fiber, (MCP): Microbial crude protein, (BW): Body weight, (BCS): Body condition score, (TP): Serum total protein, (Alb): Albumin, (Glu): Glucose, (NEFA): Non-esterified fatty acid, (BHBA): β-hydroxybutyrate, (GSH-Px): Glutathione peroxidase, (SOD): Superoxide dismutase, (TAC): Total antioxidant capacity, (MDA): Malondialdehyde, (IgG): Immunoglobulin G, (TNF-α): Tumor necrosis factor-α, (BUN): Blood urea nitrogen, (CK): Creatine Kinase, (IL-): Interleukin-, (Hsp72): Heat Shock Protein 72, (T4 and T3): Thyroid hormones, (ALT): Alanine transaminase, (RR): Respiration rate, (RT): Rectal temperature, (AI): Artificial insemination

7. Not reported

## Chromium

Our literature review of chromium’s effects on heat-stressed dairy cows identified 15 articles encompassing 16 studies, detailed in Table 1. Chromium is among the most extensively researched microminerals for mitigating HS in dairy cows. Targeted supplementation strategies, including Cr, have been explored to counteract the detrimental effects of HS, particularly by optimizing nutrient utilization and strengthening immune function. With a growing body of evidence supporting its efficacy, Cr is increasingly recognized as a key nutritional intervention for improving dairy cow health and performance in stressful production environments. However, it is important to note that the use of certain Cr compounds in dairy cow nutrition is restricted within the European Union due to potential adverse effects. For instance, the maximum safe inclusion level for Cr chelate of DL-methionine has been set at approximately 0.4 mg Cr/kg of complete feed, whereas no such restrictions currently apply to Cr picolinate [32]. These regulatory limitations may partially account for the scarcity of data on Cr supplementation in heat-stressed dairy cows reported from European countries.

### Effect of Chromium on Production Performance

The significant increase in DMI following Cr supplementation (6 out of 16 studies included; *P* ≤ 0.05) at various doses ranging from 3.1 to 62 mg/day supports its role in enhancing nutrient utilization [7, 11, 12, 18, 19, 23]. In contrast, 3 out of 16 included studies found no significant effect of Cr supplementation in the dose range of 6.2 to 24 mg/day on DMI in dairy cows subjected to HS [20, 33, 34]. These conflicting findings may stem from various factors, including differences in Cr dosage, its chemical form [35], physiological and ambient conditions, and days in milk (DIM) at the study’s onset. This underscores the need for further research and a comprehensive meta-analysis to identify potential moderators beyond Cr that may influence the observed outcomes. Han et al. [36] reported that HS can suppress the expression of the hypothalamic appetite-related peptide (Hcrt) gene, potentially disrupting the neural regulation of feed intake. This downregulation may impair appetite signaling pathways, ultimately reducing DMI. They further suggested that the upregulation of neuropeptide Y (Npy), a key regulator of energy homeostasis and a crucial mediator of stress adaptation, may serve as an anti-stress mechanism that contributes to the reduction in DMI. Given the observed effects of Cr supplementation in mitigating HS and enhancing DMI, it can be hypothesised that its efficacy may, in part, be mediated through the regulation of genes associated with HS response. This hypothesis offers a valuable direction for future research to clarify more precisely the Cr role in thermotolerance and feed intake regulation. HS conditions are frequently associated with impaired feed digestibility and reduced nutrient absorption [37]. Supplementation with inorganic Cr (0.5, 1.0, and 1.5 mg/day and 0.5, 1.0, and 1.5 mg/kg DM, respectively) has been reported to mitigate these adverse effects by enhancing organic matter digestibility in ruminants, including goats [38] and dairy buffaloes [39], ultimately leading to increased DMI. Apart from its impact on DMI, Cr supplementation (0.4 to 3 mg/day) also enhances feed efficiency in heat-stressed conditions [40, 41].

Likewise, Cr supplementation has been associated with improved milk yield across 6 out of 16 studies included (*P* ≤ 0.05) at doses ranging from 3.6 to 12.5 mg/day [7, 12, 13, 18–20]. In contrast to these findings, 4 studies reported no significant effect of Cr supplementation in the dose range of 3.1 to 62 mg/day on milk production in dairy cows experiencing HS [11, 23, 33, 42]. These conflicting findings may stem from differences in the chemical form of Cr [35], and different management systems. This variability highlights the need for further clarifying the underlying mechanisms of Cr effects on DMI under HS. The increase in milk yield observed is likely due to chromium’s role in enhancing DMI, which stimulates volatile fatty acid (VFA) production in the rumen [12, 13]. This effect supports increased milk yield by promoting propionate production, a key substrate for gluconeogenesis, thereby increasing glucose availability for lactose synthesis in mammary tissue. On the other hand, the positive effects of Cr supplementation on milk yield may be linked to improved nitrogen utilization, as indicated by lower serum urea nitrogen concentrations in heat-stressed dairy cows supplemented with 6.2 mg/day [20].

Some researchers attributed the observed increase in milk production to the elevated levels of superoxide dismutase (SOD) and glutathione peroxidase (GSH-Px) in Cr-supplemented dairy cows under HS, indicating enhanced antioxidant capacity and reduced oxidative stress [12]. Soltan [7] reported that supplementation with 6 mg/day of Cr per cow increased DMI, leading to greater energy availability and consequently higher milk yield. In addition, Zhao et al. [13] reported that the significant increase in milk yield observed with Cr supplementation is primarily attributed to an improved energy supply, facilitated by the optimal utilization of carbohydrates by rumen microbiota. However, Wang et al. [12] suggest that the effects of Cr supplementation in dairy cows under HS are entirely dose-dependent. They indicate that an optimal dose (up to 8 mg/day/cow) enhances DMI by 1.8 kg (25.8 vs. 24), while 4 mg/day/cow increases milk production by 1.5 kg (38.4 vs. 36.9), whereas excessive supplementation may have adverse effects, potentially reversing these benefits and impairing overall productivity. In contrast, Mirzaei et al. [23] reported a significant improvement in DMI of dairy cows under HS when supplemented with substantially higher Cr doses (31 and 62 mg/day per cow). Therefore, further research is required to determine the optimal dose range and more precisely elucidate the physiological pathways and mechanisms through which Cr influences milk yield under HS conditions. Notably, in addition to milk yield, studies show additional benefits, including higher fat-corrected milk (FCM), energy-corrected milk (ECM), and increased yields of milk fat, protein, lactose, and total solids, indicating that Cr not only enhances milk volume but also improves overall milk quality [12, 20, 23]. Furthermore, reductions in milk urea nitrogen (MUN) and somatic cell count (SCC) at 4 mg/day/cow suggest that Cr may enhance nitrogen utilization efficiency and improve udder health [12]. Chromium’s effects extend beyond production parameters to physiological adaptability. Several studies documented reductions in rectal temperature (RT) and respiration rate (RR), suggesting that Cr may enhance thermoregulation and mitigate HS [11, 12, 22].

### Effect on Serum Metabolites, Immune Function and Inflammatory Response

Chromium supplementation has been extensively evaluated for its influence on key serum metabolites, particularly those related to glucose-insulin homeostasis, lipid metabolism, protein metabolism, and oxidative stress. Detailed information is provided in Table 1. A notable effect of chromium is its role in regulating glucose metabolism, as evidenced by several studies reporting increased serum glucose concentrations (e.g., 3.24 vs. 3.01 and 3.75 vs. 3.39 mmol/L, within a dose range of 3.6–7.2 mg/day) and improved glucose-to-insulin ratios, which suggest enhanced feed efficiency and energy utilization [19, 20]. However, a dose-dependent response has been observed, as higher Cr levels (10.8 mg/day) were associated with decreased glucose concentrations [19], potentially due to increased insulin sensitivity and higher cellular uptake [43, 44]. Chromium enhances cellular glucose uptake by stimulating key components of insulin signaling, including IR-β kinase activity, phosphatidylinositol 3-kinase (PI3K), and protein kinase B (Akt), while promoting GLUT4 translocation to the cell surface [24]. Additionally, it mitigates insulin resistance by downregulating PTP-1B, reducing endoplasmic reticulum (ER) stress, transiently activating adenosine-monophosphate-activated protein kinase (AMPK), and facilitating cholesterol efflux from membranes, further promoting glucose uptake [24].

Likewise, evidence suggests that chromium’s influence on serum total protein (TP) concentration is dose-dependent. Supplementation with 6.6 mg/day significantly increased TP levels (7.35 vs. 7.25 g/dL), whereas a 12 mg/day dose led to a decrease (79.19 vs. 82.03 mg/dL) [18, 22]. Based on study findings, the increase in milk yield at a 12 mg/day Cr, alongside the reduction in pro-inflammatory cytokine levels [13, 18], suggests that higher Cr supplementation may redirect serum proteins toward immune enhancement and oxidative damage repair. This adaptive response likely contributes to maintaining homeostasis in dairy cows under HS conditions. Chromium supplementation at doses of 6.6 and 31 mg/day increased serum albumin (Alb) levels (3.91 vs. 3.77 and 4.12 vs. 3.92 g/dL, respectively) and increased the albumin-to-globulin ratio [18, 22, 23]. Conversely, reductions in non-esterified fatty acids (NEFA), indicated by values of 0.36 vs. 0.54 mEq/L and 144.6 vs. 158.9 µEq/L at doses of 6 and 31 mg/day, respectively, suggest reduced fat mobilization [7, 23]. The observed increase in DMI and milk yield in Cr-supplemented dairy cows under HS may provide a clearer explanation for the reduced NEFA concentrations. By mitigating negative energy balance, particularly during early lactation, Cr supplementation likely reduces adipose tissue mobilization, thereby preventing the elevation of NEFA levels in the serum.

Chromium has mixed effects on lipid metabolism, with some studies reporting increased cholesterol levels [18, 45], while others documented decreased cholesterol concentrations [42, 45]. Improvements in antioxidant enzyme activity, including increased levels of GSH-Px, SOD, and total antioxidant capacity (TAC), along with decreased malondialdehyde (MDA), support the role of chromium in mitigating oxidative stress [11, 12, 34]. Additionally, the immunomodulatory effects of Cr supplementation have been well documented, with evidence pointing to reductions in inflammatory cytokines and enhanced immune function. Studies have consistently reported significant reductions in tumor necrosis factor-α (TNF-α), IL-1β, IL-2, and IL-4 following chromium supplementation within the dose range of 3.1 to 12.5 mg/day, indicating that Cr may exert a broad anti-inflammatory effect [11, 13]. Similarly, increased IL-10 and upregulation of heat shock protein 72 (Hsp72) suggest that Cr (at 3.5 mg/day) may enhance cellular stress adaptation and immune function [45]. Chromium has been associated with increased immunoglobulin G (IgG) levels in heat stressed dairy cows, which indicates improved humoral immunity and disease resistance [11]. Chromium modulates and enhances immune function in other ruminants, as supplementation with 1 and 1.5 mg/kg of DMI increased lymphocyte proliferation, neutrophil phagocytic activity, and plasma total immunoglobulin, while reducing cortisol levels under HS [46]. Moreover, reductions in rectal temperature and respiration rate reinforce its role in stress adaptation and thermoregulation, suggesting benefits beyond direct immune modulation [12, 22]. Additionally, Cr has been linked to alterations in polymorphonuclear leukocyte counts and follicular dynamics, hinting at potential reproductive immune benefits [15].

### Effect on Ruminal Function and Microbial Activity

Chromium supplementation enhances ruminal fermentation and microbial activity, contributing to improved nutrient digestion and energy availability. In particular, several studies have documented increased concentrations of VFAs, including acetate, propionate, and butyrate, following chromium supplementation within the dose range of 4–12.5 mg/day, indicating enhanced fermentation efficiency [12, 13]. Chromium supplementation at 6.5 mg/day has been associated with favorable shifts in the ruminal microbial population, including increased abundances of *Olsenella* and *Shuttleworthia*, and reduced levels of *Escherichia-Shigella*, *Enterobacter*, and other potentially harmful genera [17]. Such microbial modifications may enhance fiber digestion, nutrient absorption, and overall production performance. These findings suggest that Cr supplementation not only supports rumen health and microbial stability but also plays a role in enhancing fermentation efficiency and nutrient utilization, ultimately benefiting dairy production.

**Fig. 3:**
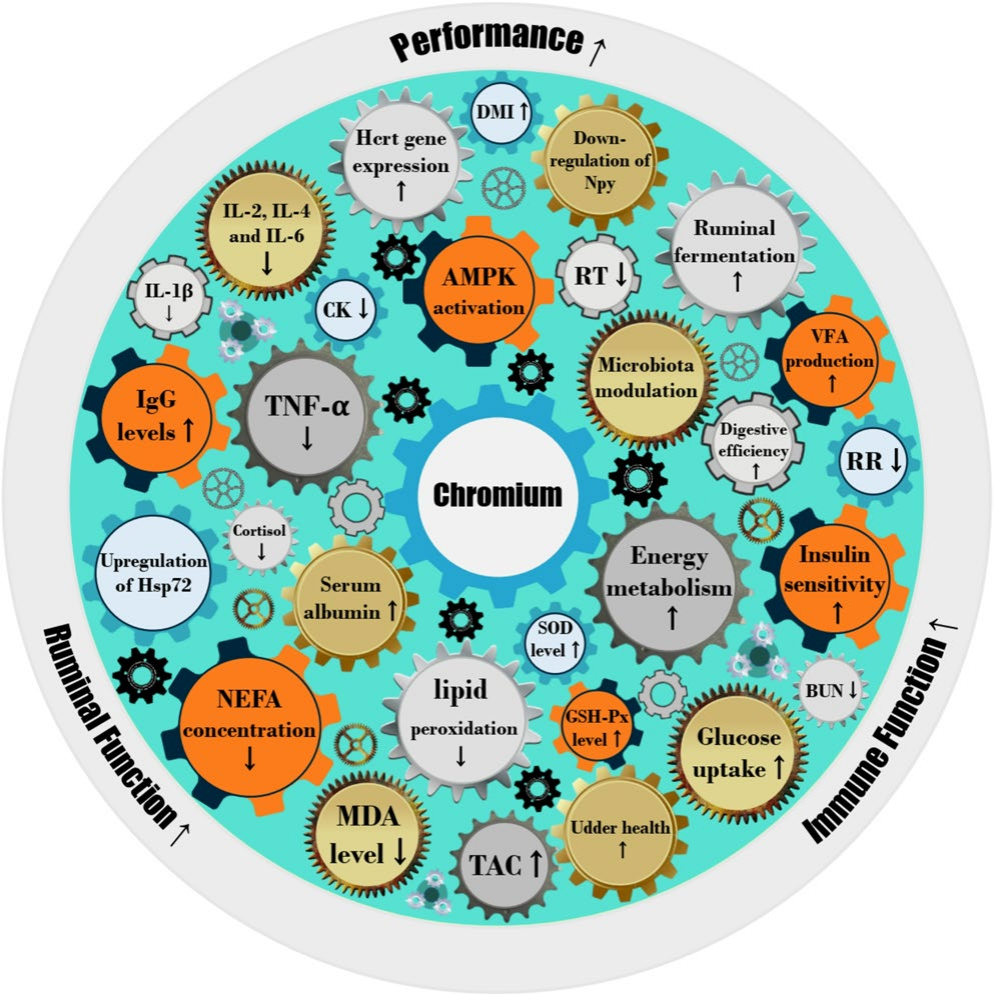
Summary of Chromium’s effects on heat-stressed dairy cows, synthesized from the included studies (Table 1). Note: The proximity of gear icons does not imply a direct relationship between variables; rather, the figure provides an overview of changes that were statistically significant (*P* ≤ 0.05). DMI: dry matter intake, VFA: volatile fatty acids, RT: rectal temperature, RR: respiration rate, AMPK: adenosine-monophosphate-activated protein kinase, BUN: blood urea nitrogen, SOD: superoxide dismutase, GSH-Px: glutathione peroxidase, MDA: malondialdehyde, TAC: total antioxidant capacity, NEFA: non-esterified fatty acids, Hsp-72: heat shock protein-72, TNF-α: tumor necrosis factor-α, IgG: Immunoglobulins G, CK: Creatine kinase, IL-: interleukin-

The collective findings from these studies underscore the significant role of Cr supplementation in enhancing dairy cow performance, metabolic health, immune function, and ruminal efficiency, particularly under HS conditions. By improving DMI, milk production, serum metabolic profiles, inflammatory responses, and ruminal fermentation, Cr emerges as a valuable dietary strategy to optimize dairy production (Fig. 3). The observed dose-dependent effects further emphasize the importance of precise supplementation strategies to maximize chromium’s benefits in dairy nutrition.

## Selenium

A comprehensive literature search on selenium’s impact on heat-stressed dairy cows identified 7 relevant articles covering 10 studies, as detailed in Table 1. Selenium is a crucial trace mineral that plays a fundamental role in antioxidant defense, immune function, and cellular stability in dairy cows. Its biological activity is primarily driven by selenoproteins, such as glutathione peroxidase, which safeguard cells against oxidative stress induced by heat exposure. Given the physiological challenges of heat stress, selenium supplementation has been widely studied as a nutritional strategy to reduce oxidative damage, support physiological stability, and improve thermotolerance, reproductive performance, and immune function in dairy cattle.

### Effect of Selenium on Production Performance, BW and BCS

Se supplementation has been widely studied for its role in improving the physiological conditions and performance of dairy cows, with evidence suggesting effects on body condition, and metabolic state. However, Se supplementation in heat-stressed dairy cows showed no significant effect on DMI or milk yield (4 and 5 studies, respectively) [10, 29–31], though slight alterations in milk composition have been reported. Two studies reported an increase in milk fat yield following Se supplementation, particularly with organic Se sources [30, 31]. The rumen plays a crucial role in producing key precursors for milk fat synthesis, making alterations in rumen fermentation a likely primary driver of increased milk fat content. Faixova et al. [47] investigated the impact of Se-yeast supplementation on rumen fermentation in lambs and reported a significant increase in alkaline phosphatase and glutamate dehydrogenase activity in rumen fluid. They attributed this effect to Se-yeast’s influence on rumen microbial composition. These findings may partially explain the observed alterations in milk fat content. Notably, as highlighted by Zheng et al. [48] in ruminants, rumen microorganisms exhibit 3.8 to 4 times greater efficiency when utilizing organic Se compared to inorganic Se, a factor that should be carefully considered in future research. Additionally, Se at 4 g/day has been linked to improved body weight (BW) and body condition score (BCS), which are crucial indicators of overall animal health and productivity [31]. The enhancement in body condition without a significant change in DMI following Se supplementation in heat-stressed dairy cows suggests a potential role of Se in optimizing feed efficiency. Moreover, supplementing heat-stressed dairy cows with 6.76 mg/day of Se-yeast led to a significant reduction in SCC (157 vs. 312) in milk [27], suggesting enhanced udder health. This result aligns with our finding, which indicate improved antioxidant status in Se-supplemented cows under HS conditions. However, some investigations reported no observable effects on production performance, physiological parameters, or rumen fermentation profiles [27, 29]. These findings highlight the potential influence of Se dosage, supplementation method, and source on its effectiveness in dairy production.

### Effect on Serum Metabolites and Antioxidant Status

Se plays a crucial role in oxidative stress regulation and metabolic balance. As shown in Table 1, two articles including four studies have demonstrated that Se supplementation increases GSH-Px activity, a key antioxidant enzyme against oxidative damage [8, 27]. This finding is both logical and expected, given that Se serves as a crucial structural component of the GSH-Px enzyme. Furthermore, two studies showed that Se reduces MDA levels, an indicator of lipid peroxidation, and enhances TAC, SOD, catalase, and glutathione levels [9, 10]. These findings confirm the significant positive impact of Se supplementation in alleviating oxidative stress in heat-stressed dairy cows. However, the underlying reasons for its lack of effect on feed intake and milk yield remain unclear, underscoring the need for larger-scale studies exploring different conditions and dosage levels.

An article involving two studies on heat-stressed Friesian dairy cows demonstrated that supplementation with 0.31 and 0.50 mg/kg DM of Se per day significantly increased NEFA concentrations while reducing beta-hydroxybutyrate (BHB) levels, which may indicate improved energy balance [8]. The elevated NEFA concentrations observed in serum align with the lack of effect of Se supplementation on DMI. The effects of Se on lipid peroxidation remain variable, with one study reporting an increase in lipid peroxidase activity [31], while others observed reductions in lipid peroxidation markers [9, 10]. As previously discussed, these conflicting findings underscore the necessity for further research to clarify the underlying mechanisms. Moreover, the supplementation of organic (0.50 mg/kg DM) and inorganic (0.31 and 0.50 mg/kg DM) Se led to a reduction in serum sodium (Na) concentrations in heat-stressed Friesian dairy cows [8].

### Effect on Immune Function, Inflammatory Response and Reproductive Performance

As shown in Table 1, Se plays a role in modulating immune responses and inflammation. In particular, two studies reported a reduction in lymphocyte counts following supplementation with Se at 0.31 and 0.50 mg/kg DM [8]. Additionally, Se has been linked to a significant reduction in cortisol levels, a key stress hormone, suggesting enhanced stress tolerance [9]. However, Kumar et al. [31] reported an increase in cortisol concentrations in heat-stressed indigenous Indian dairy cows supplemented with 4 g/day Se. This discrepancy may primarily be attributed to breed-specific differences in physiological responses to Se supplementation. Selenium also increases prolactin and triiodothyronine (T3) levels at a dose of 27.7 mg/day, which play essential roles in lactation and metabolic regulation [9]. Furthermore, based on two studies, Se supplementation at doses of 3.36 and 27.7 mg/day increased SOD, catalase, glutathione, and TAC, and decreased in MDA, hydrogen peroxide, and nitric oxide levels [9, 10]. These findings suggest that Se plays an important role in enhancing immune function and reducing oxidative stress-induced damage.

Se has been implicated in reproductive performance, particularly in relation to follicular development and artificial insemination (AI) success. One study reported that Se supplementation (27.7 mg/day) significantly increased both the number and diameter of follicles on day 10 of the estrous cycle [9]. Additionally, Se enhances progesterone levels, which are critical for maintaining pregnancy [9]. These reproductive benefits suggest that Se supplementation may improve fertility outcomes, ovarian function, and overall reproductive efficiency, making it a valuable component in dairy herd management.

**Fig. 4:**
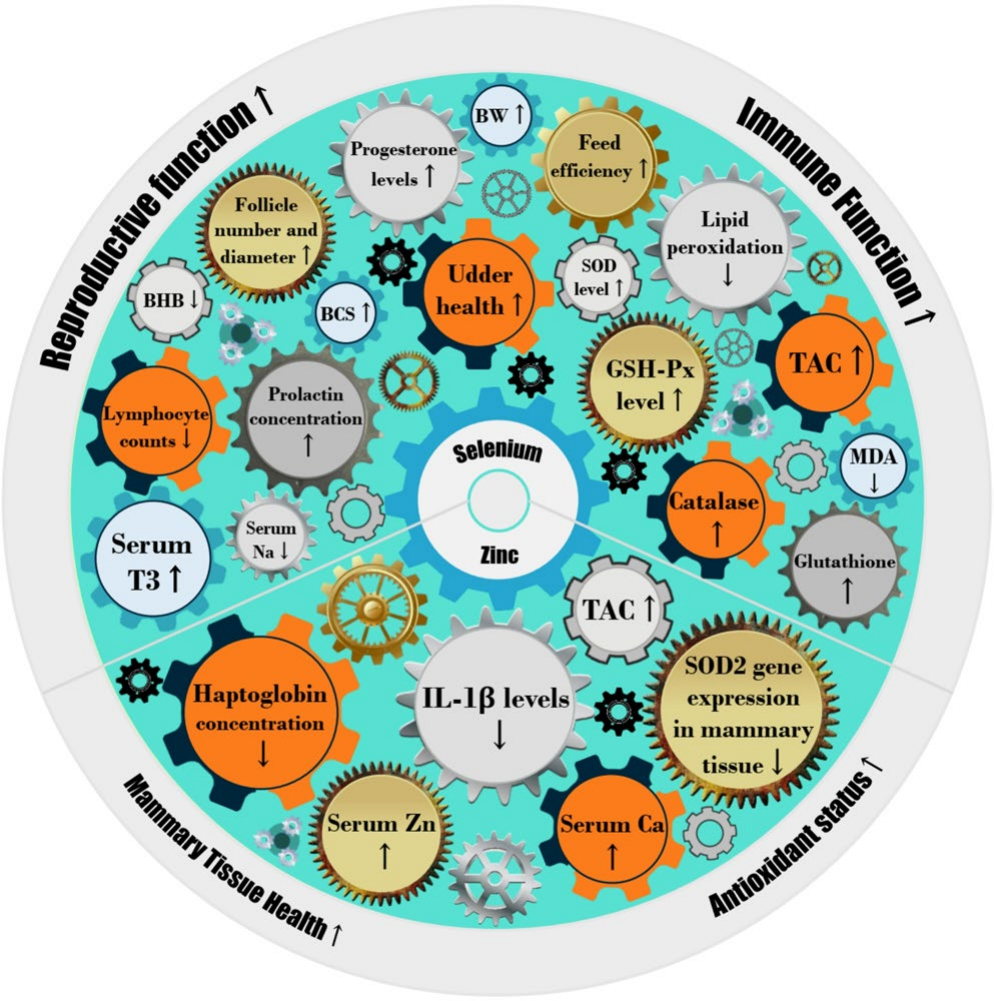
Summary of the effects of Selenium and Zinc on heat-stressed dairy cows, synthesized from the included studies (Table 1). Note: The proximity of gear icons does not imply a direct relationship between variables; rather, the figure provides an overview of changes that were statistically significant (*P* ≤ 0.05). The greater effects listed of each mineral in the figure reflect the volume of available research rather than its relative effect strength. BW: body weight, BCS: body condition score, SOD: superoxide dismutase, GSH-Px: glutathione peroxidase, MDA: malondialdehyde, TAC: total antioxidant capacity, IL-1β: interleukin-1β, Na: Sodium, Ca: Calcium, T3: Triiodothyronine, BHB: β-Hydroxybutyric acid

Overall, Se supplementation exerts significant effects on metabolic health, immune function, and reproductive efficiency. While its effects on DMI and milk production are often unremarkable and vary depending on dosage and source, Se consistently enhances antioxidant defenses, alleviates oxidative stress, and strengthens immune function. Additionally, its role in reproductive function highlights its importance in fertility management (Fig. 4). These findings suggest that optimized Se supplementation strategies can enhance dairy productivity and overall health, particularly when considering source and administration methods.

#### Zinc

Through an extensive literature review on zinc’s effects in heat-stressed dairy cows, 4 relevant articles were identified, as outlined in Table 1. Zinc has been associated with improved antioxidant capacity, reduced inflammation, and enhanced calcium homeostasis, which may contribute to better physiological adaptation to HS. Additionally, it may influence cellular integrity and metabolic processes, potentially mitigating the adverse effects of thermal stress. However, its impact on production performance outcomes remains inconclusive, warranting further research to clarify its role in heat-stressed dairy systems.

### Effect of Zinc on Production Performance

Available studies comparing different Zn sources suggest minimal differences in their effects on dairy cows under HS. Although two studies [49, 50] reported alterations in mammary tissue gene expression and serum triglyceride concentrations, these alterations did not translate into substantial improvements in production performance. Comparisons between organic and inorganic Zn sources (0.773 and 1.57 g/day per cow, respectively) revealed no significant effects on milk yield or the abundance of metabolic proteins in mammary tissues [49, 51]. The lack of significant differences may be due to insufficient baseline Zn levels in the diet, variations in bioavailability between sources, or complex interactions with other trace minerals that influence zinc’s metabolic role under HS conditions.

### Effect on Serum Metabolites, Gene Expression and Mammary Tissue Health

The influence of Zn supplementation on serum metabolites has been evaluated by two studies (Table 1). Danesh Mesgaran et al. [14] reported that supplementing heat stressed dairy cows with 2.37 g/day of organic rumen-protected Zn-Met increased serum Zn and calcium concentrations while reducing haptoglobin and IL-1β levels, indicating a potential anti-inflammatory effect. Furthermore, the study reported an increase in total antioxidant capacity, potentially contributing to improved oxidative balance in dairy cows. A comparative study of two different Zn sources, organic Zn-Met (0.773 mg/day) and inorganic Zn-hydroxychloride (1.57 mg/day), found no significant effects on the majority of serum metabolites, except for a marked increase in triglyceride concentrations in the organic Zn group [49].These findings indicate that although Zn supplementation can alter certain serum parameters, its specific effects may vary based on the source and form of Zn administered [14, 49].

The impact of Zn on gene expression and mammary tissue health in heat stressed dairy cows has been explored in two comparative studies between organic and inorganic sources. Rivas et al. [50] observed that cows supplemented with organic Zn-Met (0.773 mg/day) exhibited a decrease in SOD2 gene expression in mammary tissue compared to those receiving inorganic Zn-hydroxychloride (1.57 mg/day). Additionally, mammary cell apoptosis increased in both the epithelial and total mammary cell populations in cows receiving organic Zn-Met, suggesting potential differences in cellular turnover based on Zn source. However, Weng et al. [51] reported no significant effects of Zn source, when provided at the same doses, on overall performance or gene expression.

Overall, Zn supplementation appears to modulate certain serum metabolites, particularly increasing antioxidant capacity and altering inflammatory markers, with variations depending on the Zn source (Fig. 4). While some studies report changes in mammary gene expression and cellular turnover, the overall impact on production performance remains inconclusive. Further research is needed to elucidate the mechanistic differences between organic and inorganic Zn supplementation and their long-term implications on dairy cow productivity and health.

## Copper

Our literature search on the impact of Cu in heat-stressed dairy cows identified 2 relevant articles, the details of which are summarized in Table 1. Copper supplementation in dairy cows has been extensively studied for its potential to enhance immune function, antioxidant defense, and overall productivity; however, research conducted in dairy cows under HS is very limited. While some research highlights benefits such as improved enzyme activity and health status, responses remain inconsistent, influenced by factors such as dosage, source, and physiological conditions. Khodamoradi et al. [52] reported that administering 75 mg/day of inorganic Cu via injection on days − 40, −20, 0, and 20 relative to calving had no significant effect on overall performance, serum metabolites, or immune system indicators, except for an increase in red blood cell count at calving. Conversely, Jafari et al. [53] found that the same supplementation protocol elevated serum Cu concentrations and SOD activity by day 30 of lactation, indicating enhanced antioxidant capacity. This increase in SOD activity may be attributed to copper’s role as a cofactor for SOD, a key enzyme in oxidative stress mitigation. The discrepancies between studies may arise from variations in on-farm conditions, metabolic demands during lactation, or individual differences in Cu metabolism, underscoring the complexity of trace mineral supplementation in dairy cows and the necessity for further research.

## Cobalt

Regarding Co, our literature search identified only one article examining its effects on heat-stressed dairy cows, as detailed in Table 1. Cobalt is a vital trace mineral in dairy cows, playing a key role in rumen microbial function and vitamin B12 synthesis, both essential for energy metabolism and overall productivity. While its supplementation has been studied for potential benefits on milk production, metabolic efficiency, and health, research on its efficacy under HS conditions remains scarce, with limited studies addressing its specific impacts in such environments. Karkoodi [21] investigated the effects of inorganic Co supplementation at 30, 40, and 50 mg/day per cow, reporting increased milk yield, FCM, and milk fat yield across all supplementation levels. Notably, while 30 mg/day enhanced milk protein yield, higher doses (40 and 50 mg/day) resulted in a decline in this parameter. The observed improvements in milk and fat yields likely stem from enhanced rumen microbial activity, as Co is a key cofactor in vitamin B12 synthesis, which facilitates energy metabolism and fiber digestion. In contrast, the reduction in milk protein yield at elevated Co levels may reflect a metabolic shift favoring lipid synthesis over protein production or potential inefficiencies in microbial protein synthesis. These findings underscore the necessity for further research and the implementation of targeted Co supplementation strategies to enhance production performance while preserving milk composition.

### Multi-Microminerals

Beyond individual microminerals, our search identified a study investigating the simultaneous supplementation of multiple microminerals in heat-stressed dairy cows, with details provided in Table 1. The simultaneous supplementation of multiple microminerals has been investigated as a strategy to enhance performance, reproductive efficiency, and metabolic balance in dairy cows. However, research on its effectiveness under HS conditions remains scarce. The combined effects of essential trace minerals may contribute to improved physiological adaptability, yet responses are influenced by nutrient interactions, bioavailability, and metabolic demands. Khorsandi [16] et al. examined the impact of a sustained-release multi-trace element and vitamin bolus, delivering daily mean nutrient release rates of 136.5 mg Cu, 2.1 mg Se, 2.0 mg Co, 4.1 mg I, 69.4 mg Mn, and 111.5 mg Zn. Supplementation resulted in significant improvements in milk composition, including increased milk fat, protein, and solids-not-fat (SNF) content, while reducing milk SCC. Additionally, elevated serum total protein levels at calving suggested improved metabolic and immune status. Reproductive performance was also improved as reflected by a decrease in days open and an increase in cumulative pregnancy rate by the fifth AI. These benefits may be attributed to the synergistic role of microminerals in antioxidant defense, immune function, and enzymatic activities essential for lactation and fertility. These findings underscore the potential advantages of multi-micromineral supplementation in dairy cows under HS, warranting further research to optimize formulations and evaluate long-term impacts.

## Conclusion

In conclusion, micromineral supplementation presents a promising strategy for mitigating the detrimental effects of HS in dairy cattle by supporting metabolic efficiency, immune function, and oxidative balance. While Cr enhances energy metabolism and feed intake, Se plays a pivotal role in antioxidant defense and immune modulation. Zinc, Cu, and Co contribute to cellular stability, enzymatic function, and overall productivity, though their effectiveness varies based on dosage, bioavailability, and dietary interactions. Multi-micromineral supplementation offers synergistic benefits, improving milk composition, udder health, and reproductive performance under thermal stress. However, inconsistencies in research findings underscore the need for further investigations to refine supplementation protocols and optimize mineral balance for enhanced productivity in heat-stressed dairy systems.

## Data Availability

No datasets were generated or analysed during the current study.
